# Clinico-psychopathological characteristics of patients with residual states in long-term schizophrenia

**DOI:** 10.1192/j.eurpsy.2023.2319

**Published:** 2023-07-19

**Authors:** V. Mitikhin, M. Kuzminova

**Affiliations:** Department of Mental Health Support Systems Research Centre, Mental Health Research Centre, Moscow, Russian Federation

## Abstract

**Introduction:**

Long-term schizophrenia, even in remission, is necessarily accompanied by residual symptoms that may be quite pronounced and may significantly affect many aspects of the patient’s life, requiring exploration of specific therapeutic approaches. The alleviation of residual symptoms is an important factor in the patient’s better adjustment.

**Objectives:**

Assessment and study clinical characteristics long-term schizophrenia.

**Methods:**

Clinical, statistical, psychometric. A total of 90 patients, mean age 66,6±13,3 years, 26 males, 64 females were examined.

**Results:**

Negative symptoms were predominant in patients with long-term schizophrenia (17,8±6,7). It was represented by: abstract thinking disorders (2,8±1,0), stereotyped thinking (2,7±1,1), passive-apathetic social isolation (2,6±1,2), avolition (2,6±0,8), flattening of affect (2,5±0,8). It manifested as lack of expressiveness in facial expressions and gestures, deficit of communicative gestures as well as emotional indifference (2,4±1,1), limitation of contacts with people, and spontaneous and fluent speech impairments. Positive symptoms were rare, mainly represented by suspiciousness (2,2±1,2), sometimes rising to delirium (1,8±1,4). Conceptual disorganization was detected in 1,9±0,7. Agitation and aggression were generally not characteristic of those surveyed. Depression/anxiety was quite pronounced in patients with long-term schizophrenia. Depression (1,8±0,8) was represented by low mood, hopelessness and loss of social interests. Anxiety (2,9±1,2) was even more prominent and predominant amongst all symptoms.

**Conclusions:**

The authors expanded our understanding of the clinical characteristics of residual symptoms of long-term schizophrenia to allow timely identification and provision of medical and rehabilitative care.

**Disclosure of Interest:**

None Declared

